# Knowledge Level of Cardiovascular Disease Risk Factors and Sleep Quality in People With Epilepsy

**DOI:** 10.1002/brb3.71573

**Published:** 2026-07-08

**Authors:** Heycan Erdoğan, Hatice Karabuğa Yakar

**Affiliations:** ^1^ Institute of Health Sciences Marmara University Istanbul Türkiye; ^2^ Faculty of Health Sciences Marmara University Istanbul Türkiye

**Keywords:** cardiovascular disease, epilepsy, sleep quality

## Abstract

**Background:**

Knowledge level about sleep quality and cardiovascular disease risk factors in people with epilepsy is an important but insufficiently researched area.

**Objective:**

This study was conducted to determine the knowledge level about cardiovascular disease risk factors and the sleep quality of people with epilepsy. This descriptive study was conducted between January and April 2024 with 303 individuals diagnosed with epilepsy. Data were collected using the Individual Descriptive Characteristics Form, Cardiovascular Disease Risk Factors Knowledge Level Scale (CARRF‐KL), and Pittsburgh Sleep Quality Index (PSQI). Independent groups *t*‐test, one‐way analysis of variance, post hoc tests correlation, and linear regression analyses were used in the data analysis.

**Results:**

The mean age of the patients was 41.84 ± 11.92, 80.2% having been diagnosed with epilepsy for more than 10 years, and 86.8% diagnosed with generalized epilepsy. The mean CARRF‐KL total score of people with epilepsy was 19.40 ± 3.09, and their knowledge level was above average. The mean total score of PSQI was 6.44 ± 4.32, suggesting poor sleep quality. The total score of cardiovascular disease risk factors knowledge level of individuals diagnosed with epilepsy differed according to gender, marital status, epilepsy type, and presence of additional chronic diseases (*p* < 0.05).

**Conclusion:**

Half of the people with epilepsy had poor sleep quality. Knowledge level of cardiovascular disease risk factors was above average.

## Graphical abstract

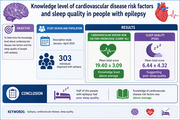

## 1 Introduction

Epilepsy is a global neurological health issue that affects 50 to 70 million people in low‐ and middle‐income countries, impacting them sociologically, culturally, and economically (Loureiro Fialho et al. 2024; Doege et al. 2021). According to the Turkish Neurology Association's 2020 data, the number of individuals diagnosed with epilepsy is approximately 750,000 in our country (World Epilepsy Day, 2020). Given the increasing frequency of epilepsy diagnoses, it is crucial to closely monitor people with epilepsy (PWE) and examine comorbid conditions. Recent studies have raised concerns about cardiac involvement in PWE, pointing out that sudden death rates are increasing in PWE due to cardiovascular complications. Cardiovascular‐related deaths account for a quarter of the mortality rate in PWE (Mayer et al. 2024). Epileptic seizures, the disease itself, and the anti‐seizure medications used can lead to cardiac complications (Surges et al. 2021). The relationship between cardiovascular disease and epilepsy is complex. Catecholaminergic fluctuations and hypoxemia associated with seizures alter myocardial and coronary vessel structures. Over time, these cardiotoxic effects lead to mechanical and electrical dysfunction, causing arrhythmias and sudden cardiac death (Verrier et al. 2020). Verrier et al. (2020) described this negative effect of epilepsy on the heart as an “epileptic heart”. Anti‐seizure medications are reported to contribute to atherosclerosis, increase vascular risk, and lead to sudden cardiac death associated with coronary events due to increases in serum lipid levels, homocysteine, and inflammatory markers (Hookana et al. 2016).

Studies have shown that the frequency of heart failure increases in PWE (Doege et al. 2021) and that they have a high risk of cardiovascular events within five years after a seizure (Bucci et al. 2023). In the study by Doege et al. (2021), among a group of approximately 9600 patients with and without epilepsy who were followed for ten years, heart failure was diagnosed in 28.6% of patients with epilepsy and 20.4% of patients without epilepsy; while in the study by Bucci et al. (2023), it was emphasized that the risk of cardiovascular disease increased over a five‐year period in approximately 15,000 individuals with epilepsy who experienced a cardiovascular event within 30 days of a seizure. The danger ratio for ischemic heart disease was reported as 3.23.

Terman et al. (2021) also emphasized that hypertension, diabetes, and coronary artery disease are more common in PWE compared to those without epilepsy. The same study, which followed approximately 17,000 individuals for five years, highlighted that 154 of them were diagnosed with epilepsy, and that 52% of those with epilepsy had a risk of atherosclerotic disease; however, the small sample size constitutes a limitation of the study.

Similarly, Lambert et al. reported that PWE are at two to three times higher risk of sudden cardiac death due to cardiac arrhythmias compared to the general population (Lamberts et al. 2015). In a retrospective study involving a sample of young individuals, ECG recordings of 185 individuals with epilepsy and 178 individuals without epilepsy were examined, and it was reported that individuals with epilepsy had a higher heart rate and QT interval prolongation compared to those without epilepsy (Lamberts et al. 2015).

Mintzer et al. (2020) and Gaertner et al. (2024) found that enzyme‐inducing anti‐seizure medications increase the risk of hyperlipidemia by 22%. In the study by Mintzer et al. (2020), it was emphasized that among 11,374 adult patients with no prior history of hyperlipidemia or previous treatment with lipid‐lowering drugs who were newly treated with anticonvulsants, 8778 (77%) were given non‐inducing drugs and 2596 were given inducing drugs, and new hyperlipidemia diagnoses were seen in 14.6% of patients who started inducing anticonvulsants and 10.7% of patients who started non‐inducing anticonvulsants.

Hookana et al. (2016) and Wang et al. (2023) noted that carbamazepine, sodium channel blockers, and valproic acid further increase the occurrence of cardiac arrhythmias and long‐term atrial fibrillation. In the study by Wang et al., 2699 individuals with epilepsy were followed for approximately 10 years, and cardiac arrhythmias developed in nearly 300 patients due to the use of anti‐seizure drugs.

Epileptic seizures often exhibit circadian patterns; different seizure types occur more frequently at certain times of the day. Circadian rhythms, regulated by the body's internal clock located in the suprachiasmatic nucleus, influence many physiological processes, including neuronal excitability and the sleep‐wake cycle. Insomnia is common among PWE (Vendrame et al. 2013), with an estimated prevalence of 50% (Gutter et al. 2019). One third of people with epilepsy report having sleep problems (Bostan and İlhan Algın 2024). Sleep deprivation disrupts circadian rhythms and increases the risk of seizures. This is particularly concerning for patients who experience nocturnal seizures, as they are more likely to have seizures following periods of sleep deprivation. Sudden unexpected epileptic death is a major concern for people with epilepsy, and emerging evidence suggests that it may affect the occurrence of circadian rhythms (Niu et al. 2025).

Circadian rhythms regulate numerous physiological processes, including heart rate variability, respiratory patterns, and autonomic function. Cardiac rhythm disturbances such as bradycardia, asystole, and arrhythmias have been observed in epileptic patients, particularly during sleep and nocturnal seizures. These disorders are modulated by circadian effects on parasympathetic nervous system activity, which is usually dominant at night.

In epilepsy patients, the autonomic nervous system may already be impaired, leading to increased susceptibility to cardiac events following seizures. Changes in sleep‐related respiratory control, particularly the body's decreased sensitivity to CO2 levels during Non‐Rapid Eye Movement (NREM) sleep, can worsen during seizures and increase the risk of fatal outcomes. Therefore, circadian rhythms affect both the cardiovascular and respiratory systems, and disruptions to these during seizures can lead to sudden, unexpected death (Niu et al. 2025).

Epilepsy patients frequently experience autonomic symptoms during seizures, including changes in heart rate, respiration, gastrointestinal, urogenital, and pupillary function. Rapid Eye Movement (REM) sleep disorder and nocturnal seizures are common in patients with epilepsy. Night shifts lead to increased sympathetic activity, decreased heart rate variability, and impaired blood pressure. Poor sleep creates imbalances in the autonomic nervous system, increasing the risk of cardiovascular disease (Niu et al. 2025; Shao et al. 2025). Autonomic dysfunction can affect blood pressure, heart rate, and cardiac rhythm control, creating long‐term cardiovascular stress and risk factors. İnsomnia can further increase this risk by enhancing sympathetic activation (Hamdy et al. 2022).

Sleep disorders make seizure control difficult (Çilliler and Güven 2020; Moore et al. 2021; Quigg et al. 2016). As the frequency of seizures increases, sleep quality worsens (Nobili et al. 2021), and patients experience daytime sleepiness (Çilliler and Güven 2020). Therefore, when evaluating cardiovascular disease risk in PWE, it is essential also to assess sleep health.

When examining the lifestyle behaviors of individuals with epilepsy who are at risk of cardiovascular disease, it is observed that they do not exercise regularly, do not pay attention to their diet, and are obese, indicating that they do not exhibit behaviors appropriate for cardiovascular disease risk factors. This lifestyle behavior also affects the patients' sleep quality.

Exercise is protective against many health conditions, including cardiovascular disease, diabetes, and mental health. Unfortunately, people with epilepsy engage in less physical activity than the general population, and the reasons for this include a variety of psychosocial and clinical factors such as lack of time, transportation problems, accidents, injuries, fear of triggering seizures, and the stigma associated with participating in team or group sports (Chang et al. 2025; Mueller et al. 2024).

In a study examining the dietary habits of nearly one hundred people with epilepsy, it was reported that people with epilepsy exhibited more unfavorable dietary behaviors compared to healthy people; they consumed plant‐based products such as fruits, legumes, seeds, and nuts, which are sources of vitamins, minerals, and dietary fiber, significantly less frequently (Szałwińska et al. 2021). A study conducted in Malaysia indicated that approximately 40% of 166 people with epilepsy were obese (Chang et al. 2025); a meta‐analysis study conducted on a total of 17 clinical trials involving 5329 epilepsy patients indicated that epilepsy patients are more prone to obesity than healthy individuals (Li et al. 2024). Exercise reduces sleep problems in people with epilepsy (Eriksen et al. 1994; Shawahna and Abdelhaq 2020); obesity can trigger sleep problems in people with epilepsy (Söylemez et al. 2020).

Therefore, it appears that cardiovascular disease risk factors have components that affect sleep. It is thought that people with epilepsy have low awareness of their risk of developing cardiovascular disease and are unable to apply this awareness into behavioral changes.

The interaction between epilepsy, epileptic seizures, sleep, and cardiac function is an important but understudied area of research. People with epilepsy have the right to a healthy and quality life, which is among the sustainable development goals of our country. Identifying sleep problems in patients and to determine the level of knowledge of cardiovascular disease risk factors in people with epilepsy at an early stage and planning interventions will have a positive impact on patients’ quality of life and prevent sudden deaths. No study has been found, either in our country or abroad, that simultaneously evaluates the cardiovascular disease risk factors knowledge level and sleep quality in PWE.

Based on this gap, the current study was conducted to determine the level of knowledge about cardiovascular disease risk factors and sleep quality in PWE.

*Research Questions*

**S_1_**: What is the level of knowledge of PWE about cardiovascular disease risk factors?
**S_2_**: What is the sleep quality like in PWE?
**S_3_**: Does the level of knowledge about cardiovascular disease risk factors differ according to the descriptive characteristics of PWE?
**S_4_**: Does sleep quality differ according to the descriptive characteristics of PWE?

## 2 Methods

### 2.1 Study Design

This is a descriptive study.

### 2.2 Setting

The study was conducted between January and April 2024 with patients who were followed up with a diagnosis of epilepsy in the Neurology Outpatient Clinic and Neurology Subspecialty Outpatient Clinic (Epilepsy Outpatient Clinic) of a Training and Research Hospital located on the European Side of Istanbul, Turkey.

### 2.3 Participants

Patients who have been followed up with an epilepsy diagnosis for at least one year, have no communication problems, are literate, have agreed to participate in the study, and were included in the study. Patients who incompletely filled out the data collection forms were excluded.

### 2.4 Sample Size

**FIGURE 1:**
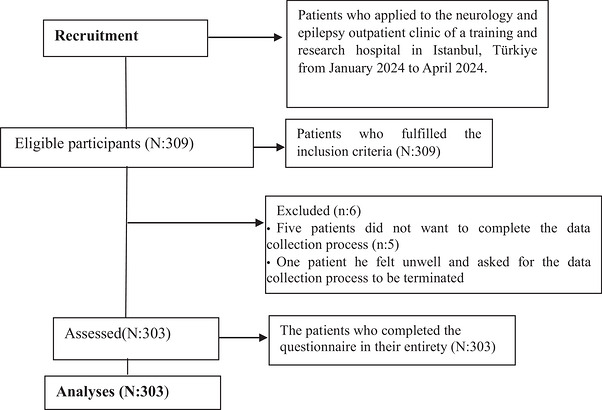
Flowchart for patients’ recruitment process.

The sample size of the study was determined using a priori power analysis. Sample size calculation was made for the regression analysis test as 107 by taking an effect size *f* ^2^ 0.15, 5% margin of error (*α* = 0.05), and 95% power (1 – *β*) = 0.95 (Faul et al. 2007). A total of 309 patients were reached, but the study was completed with 303 people with epilepsy who met the inclusion criteria and completed the data collection forms without missing information. The patient participation process is summarized in Figure 1.

The diagnostic criteria for epilepsy patients included in the sample were established based on the physician's knowledge and experience. Epilepsy was diagnosed based on video recordings of seizures and descriptions of seizures provided by patients and their relatives. Patients diagnosed were assigned the ICD code G40. G40.0 represents focal idiopathic epilepsy; G40.1 represents focal symptomatic epilepsy; G40.2 represents symptomatic epilepsy; G40.3 represents generalized idiopathic epilepsy and epileptic syndromes; and G40.4 represents generalized epilepsy and epileptic syndromes.

### 2.5 Ethical Principles

The research was conducted after obtaining approval from the pertinent ethics review board (17.10.2023 /99). Written permission was obtained from the ethics committee of the Training and Research Hospital where the study was conducted. Before data collection began, the purpose of the study, that the information would be used for scientific purposes, and that confidentiality would be maintained were explained verbally to the potential participants. After verbal information, written and verbal consents were obtained from those who volunteered to participate in the study through the ‘Informed Subject Consent Form.’ Scientific principles and universal ethical principles were adhered to while conducting the research. Since the study involved using human subjects and protecting individual rights, the study adhered to the Helsinki Declaration of Human Rights throughout the research process.

### 2.6 Data Collection

The data for the research were collected through face‐to‐face interviews conducted by the researcher in a designated room at the involved outpatient clinics of the hospital, with people diagnosed with epilepsy. After providing information about the research (including the purpose of the study, confidentiality, where the results will be used, potential benefits or harms, etc.), interviews were conducted with people who agreed to participate in the study. Participants were informed that they could terminate their participation at any time, and verbal consent was obtained before starting the research, followed by written consent. Completing the data collection tools for each PWE took an average of 15–20 min.

### 2.7 Measurements

#### 2.7.1 The Individual Descriptive Characteristics Form

The Individual Descriptive Characteristics Form was prepared by the researcher based on the literature (Katsiki et al. 2014; Silvani 2019) and included a total of 18 questions about descriptive characteristics and disease‐related characteristics of the individual.

#### 2.7.2 The Cardiovascular Disease Risk Factors Knowledge Level Scale

It was developed by Arıkan et al. (2009), and its validity and reliability study was conducted. The scale consists of 28 items in total. The first four items assess the knowledge level about the characteristics of cardiovascular diseases, 15 items assess the knowledge level about risk factors, and 9 items assess the knowledge level about changes in risk behaviors. In the evaluation, one point is given for each correct answer. Items numbered “11‐12‐16‐17‐24‐26” are reverse‐coded. The lowest possible score on the scale is zero, and the highest score is 28. The higher the score, the higher the level of knowledge. In the study by Arıkan et al. (2009), the reliability coefficient of the scale was found to be 0.77. In this study, the reliability coefficient was 0.82, indicating that the reliability of the scale is good.

#### 2.7.3 Pittsburgh Sleep Quality Index (PSQI)

PSQI was developed by Buysse et al. (1989). The Turkish validity and reliability of the scale were tested by Ağargün et al. (1996). The PSQI provides information about sleep quality in the last month and the type and severity of sleep disturbance. The scale consists of 24 questions; 19 items are included in the scoring. Each item in the scale takes a value between 0 (no distress at all) and 3 (severe distress) points. The total score for each of the seven subscales gives the total PSQI score, which ranges from 0 to 21. A total PSQI score of ≤5 indicates “good sleep quality,” and >5 indicates “poor sleep quality.” The reliability coefficient of the scale was found to be 0.80 (Ağargün et al. 1996). In this study, the reliability coefficient was 0.84, indicating that the reliability of this scale is good.

### 2.8 Statistical Analysis

The data obtained in the research were evaluated using the IBM SPSS Statistics for Windows, Version 22.0 (SPSS INC., Chicago, IL, USA) statistical program in a computer environment. Frequency and percentage analyses were used to determine the descriptive characteristics of the participants, while mean and standard deviation statistics were used in the analysis of the scale. Kurtosis and Skewness values were analyzed to determine whether the research variables followed a normal distribution. Independent group *t*‐tests, one‐way analysis of variance (ANOVA), and post hoc (Tukey, LSD) analyses were used to examine the difference in the scale total score according to the patients’ descriptive characteristics. Cohen (*d*) coefficient was used to calculate the effect size. The effect size indicates whether the difference between the groups is large enough to be considered significant. Cohen's value is interpreted as: “0.2: small; 0.5: medium; 0.8: large,” and *r* value “< 0.2: weak correlation, < 0.5: moderate correlation, > 0.8: strong correlation” (Sullivan and Feinn 2012).

Regression analyses were conducted using R software (Version 4.3.1) with the rms package for ordinary least squares regression modeling (Harrell 2023); ggplot2 for data visualization (Wickham, 2016); car for diagnostic tests (Fox and Weisberg 2019); and lmtest for hypothesis testing (Zeileis and Hothorn 2002). Standard multiple regression analysis was employed to examine the relationship between cardiovascular disease risk factor knowledge and sleep quality, while controlling for demographic and clinical covariates (Tabachnick and Fidell 2019).

## 3 Results

The patients had a mean age of 41.84 ± 11.92 years (min = 19; max = 76), and 57.8% were female. 80.2% of the patients had been diagnosed with epilepsy for more than 10 years. 86.8% had generalized type epilepsy. The mean body mass index (BMI) of the patients was 26.45 ± 4.68 (min = 19; max = 39). 19.5% had chronic diseases other than epilepsy. Among those with chronic diseases, 50.8% had diabetes mellitus. 47.2% of the patients used levetiracetam, 36% used carbamazepine, and 29% used sodium valproate. Over half of the patients had not had a seizure in the last month (Table 1).

**TABLE 1:** Distribution of people with epilepsy according to descriptive characteristics (*N* = 303).

Descriptive characteristics	Frequency (*n*)	Percentage (%)
**Age mean (mean ± SD)**	41.84 ± 11.92 (min = 19; max = 76)	
**Gender**		
Female	175	57.8
Male	128	42.2
**Marital status**		
Married	169	55.8
Single	134	44.2
**Educational status**		
Illiterate/literate	18	5.9
Primary/secondary school	143	47.2
High school	83	27.4
University	59	19.5
**Smoking status**		
Yes	72	23.8
No	231	76.2
**Body mass ındex**		
Normal weight (18.5–24.9 kg/m^2 ^)	133	43.9
Overweight (25–29.9 kg/m^2^)	105	34.7
Obese (30 and above)	65	21.4
**Body mass index average (mean ± SD)**	26.45 ± 4.68 (min = 19; max = 39)	
**Presence of disease other than epilepsy**		
Yes	59	19.5
No	244	80.5
**Diseases other than epilepsy (*n* = 59)** ^*^		
Diabetes	30	50.8
Hyperthyroidism	15	25.4
Asthma	10	16.9
Chronic kidney disease	6	10.2
Rheumatism	5	8.5
Family Mediterranean fever	3	5.1
Multiple sclerosis	1	1.7
**Drugs used (*n* = 303)** ^*^		
Levetirasetam	143	47.2
Karbamazepin	109	36.0
Sodyum Valporat	88	29.0
Lamotrijin	40	13.2
Lakozamid	33	10.9
Okskarbazepin	27	8.9
Topiramat	21	6.9
Fenitoin	11	3.6
**Epilepsy diagnosis time**		
1–5 year	22	7.3
6–10 year	38	12.5
More than 10 years	243	80.2
**Epilepsy type**		
Generalized	263	86.8
Fokal	40	13.2
**Number of seizures in the last month**		
0 (Geçirmeyen)	182	60.1
1–3	59	19.5
3–5	35	11.5
More than five	27	8.9

^*^More than one option is marked.

The total mean score of the Cardiovascular Disease Risk Factors Knowledge Level Scale for epileptic individuals was 19.40 ± 3.09 (Table 2), and the total mean score of the Pittsburgh Sleep Quality Index was 6.44 ± 4.32 (Table 3). The total and subscale scores of the Cardiovascular Disease Risk Factors Knowledge Level Scale varied according to patient's gender (*p* = 0.02; *d* = 0.27), marital status (*p* = 0.009; *d* = 0.30), comorbidities other than epilepsy (*p* = 0.008; *d* = 0.38), and epilepsy type (*p* = 0.03; *d* = 0.35) (*p* < 0.05) (Table 4). PSQI scores did not differ according to the patients’ descriptive characteristics (Table 5).

**TABLE 2:** Distribution of findings related to the mean scores of Cardiovascular Diseases Risk Factors Knowledge Level Scale of people with epilepsy (*N* = 303).

Scale	*N*	Mean	SD	Min	Max
**Cardiovascular Disease Risk Factors Knowledge Level Total Score**	303	19.40	3.09	7.00	27.00
Cardiovascular disease characteristics knowledge level sub‐dimension	303	2.26	1.12	0.00	4.00
Risk factors knowledge level sub‐dimension	303	9.98	1.96	3.00	14.00
Change in risk behaviors knowledge level sub‐dimension	303	6.22	1.23	1.00	9.00

Min: minimum, Max: maximum, SD: standard deviation.

**TABLE 3:** Distribution of Pittsburgh Sleep Quality Scale Total Scores of people with epilepsy (*N* = 303).

Index	Frequency (*n*)	Percentage (%)	Mean	SD	Min.	Max	Index min–max score
**Pittsburgh Sleep Quality Index Total Scores**			6.44	4.32	0.00	14.00	0–21
**Good sleep quality**	136	44.9					
**Bad sleep quality**	167	55.1					

Min: minimum, Max: maximum, SD: standard deviation.

**TABLE 4:** Distribution of findings related to the comparison of Cardiovascular Diseases Risk Factors Knowledge Level Scale scores with the descriptive characteristics of people with epilepsy (*N* = 303).

Descriptive characteristics	*n*	Cardiovascular disease risk factors knowledge level total score	Cardiovascular disease characteristics knowledge level sub‐dimension	Risk factors knowledge level sub‐dimension	Change in risk behaviors knowledge level sub‐dimension
Age		Mean ± SD	Mean ± SD	Mean ± SD	Mean ± SD
30 and below	60	19.61 ± 3.46	2.51 ± 1.12	9.83 ± 1.89	6.35 ± 1.41
31–40	83	18.97 ± 3.32	2.06 ± 1.08	9.86 ± 2.25	6.12 ± 1.16
41–50	79	19.17 ± 2.40	2.31 ± 1.12	9.81 ± 1.78	6.10 ± 1.06
51–60	59	20.17 ± 2.89	2.32 ± 1.10	10.42 ± 1.92	6.45 ± 1.08
61 and above	22	19.27 ± 3.66	2.00 ± 1.19	10.22 ± 1.68	6.09 ± 1.74
*F* =		1.49	1.84	1.13	1.09
*p* =		0.20	0.11	0.33	0.35
**Gender**					
Female	175	19.76 ± 2.88	2.29 ± 1.13	10.12 ± 1.89	6.40 ± 1.19
Male	128	18.93 ± 3.31	2.21 ± 1.11	9.78 ± 2.06	5.98 ± 1.24
*t* =		2.32	0.60	1.50	2.94
*p* =		0.02*	0.54	0.13	0.004**
*d* =		**0.27**			**0.34**
**Marital status**					
Married	169	19.71 ± 2.89	2.28 ± 1.06	10.24 ± 1.89	6.24 ± 1.18
Single	134	19.03 ± 3.30	2.23 ± 1.19	9.64 ± 2.02	6.202 ± 1.29
*t* =		1.90	0.34	2.63	0.28
*p* =		0.05	0.72	0.009**	0.77
*d* =				**0.30**	
**Educational status**					
Illiterate/literate	18	18.50 ± 3.60	2.33 ± 3.60	9.44 ± 2.28	5.77 ± 1.30
Primary/secondary school	143	19.28 ± 2.80	2.16 ± 2.80	9.89 ± 1.89	6.25 ± 1.09
High school	83	19.55 ± 3.28	2.30 ± 3.28	10.09 ± 1.96	6.22 ± 1.33
University	59	19.78 ± 3.34	2.42 ± 3.34	10.18 ± 2.07	6.27 ± 1.35
*F* =		0.93	0.93	0.84	0.85
*p* =		0.42	0.42	0.47	0.46
**Smoking status**					
Yes	72	19.12 ± 3.06	2.33 ± 1.07	9.73 ± 1.97	6.13 ± 1.20
No	231	19.49 ± 3.10	2.24 ± 1.13	10.05 ± 1.96	6.25 ± 1.24
*t* =		−0.89	0.59	−1.20	−0.67
*p* =		0.37	0.55	0.22	0.50
**Body mass index**					
Normal weight	133	19.31 ± 3.27	2.18 ± 1.18	9.92 ± 2.02	6.25 ± 1.28
Overweight	105	19.50 ± 3.14	2.25 ± 1.07	10.02 ± 1.96	6.30 ± 1.21
Obese	65	19.44 ± 2.65	2.43 ± 1.07	10.01 ± 1.88	6.03 ± 1.14
*F* =		0.11	1.02	0.09	1.07
*p* =		0.89	0.36	0.91	0.34
**Epilepsy type**					
Generalized	263	19.51 ± 3.00	2.23 ± 1.14	10.04 ± 1.97	6.281 ± 1.18
Fokal	40	18.72 ± 3.60	2.45 ± 0.93	9.52 ± 1.92	5.850 ± 1.47
*t* =		1.50	−1.12	1.57	2.07
*p* =		0.13	0.26	0.11	**0.03**
*d* =					**0.35**
**Presence of disease other than epilepsy**					
Yes	59	20.27 ± 2.91	2.61 ± 1.16	10.28 ± 1.95	6.44 ± 1.14
No	244	19.20 ± 3.10	2.18 ± 1.10	9.90 ± 1.96	6.17 ± 1.24
*t* =		2.40	2.66	1.34	1.50
*p* =		0.01*	0.008**	0.18	0.13
*d* =			**0.38**		
**Epilepsy diagnosis time**					
1–5 year	22	19.63 ± 3.48	2.40 ± 3.48	9.72 ± 1.85	6.50 ± 1.43
6–10 year	38	19.36 ± 3.90	2.34 ± 3.90	9.92 ± 2.50	6.15 ± 1.30
More than 10 years	243	19.39 ± 2.92	2.23 ± 2.92	10.01 ± 1.88	6.21 ± 1.20
*F* =			0.06	0.23	0.62
*p* =			0.93	0.79	0.53

SD: standard deviation; *F*: ANOVA test; *t*: independent groups *t* test. *p < 0.05; **p < 0.001;

**TABLE 5:** Distribution of findings related to the comparison of Pittsburgh Sleep Quality Scale Total Scores according to the descriptive characteristics of people with epilepsy (*N* = 303).

Descriptive characteristics	*n*	Pittsburgh Sleep Quality Index Total Score
**Age**		**Mean ± SD**
30 and below	60	6.88 ± 4.65
31–40	83	6.44 ± 4.12
41–50	79	5.82 ± 4.60
51–60	59	6.71 ± 3.93
61 and above	22	6.81 ± 4.23
*F* = 0.65; *p* = 0.62		
**Gender**		**Ort ± SS**
Female	175	6.45 ± 4.12
Male	128	6.43 ± 4.60
*t* = 0.03; *p* = 0.96		
**Marital status**		**Ort ± SS**
Married	169	6.32 ± 4.40
Single	134	6.61 ± 4.23
*t* =		−0.58
*p* =		0.56
**Educational status**		**Ort ± SS**
Illiterate/literate	18	6.66 ± 3.91
Primary/secondary school	143	6.79 ± 4.37
High school	83	6.39 ± 4.56
University	59	5.61 ± 3.93
*F* =		1.06
*p* =		0.36
**Smoking status**		**Ort ± SS**
Yes	72	6.19 ± 4.75
No	231	6.52 ± 4.19
*t* =		−0.57
*p* =		0.59
**Body mass index**		**Ort ± SS**
Normal weight	133	6.65 ± 4.26
Overweight	105	6.21 ± 4.30
Obese	65	6.41 ± 4.52
*F* =		0.31
*p* =		0.73
**Presence of disease other than epilepsy**		**Ort ± SS**
Yes	59	5.72 ± 4.46
No	244	6.62 ± 4.28
*t* =		−1.42
*p* =		0.15
**Epilepsy diagnosis time**		**Ort ± SS**
1–5 year	22	5.77 ± 4.05
6–10 year	38	5.97 ± 4.53
More than 10 years	243	6.58 ± 4.32
*F* =		0.61
*p* =		0.54
**Epilepsy type**		**Ort ± SS**
Generalized	263	6.38 ± 4.32
Fokal	40	6.85 ± 4.34
*t* =		−0.62
*p* =		0.53

*F*: ANOVA test; *t*: independent groups *t* test.

According to the results of the multiple regression analysis, when examining whether the level of knowledge about cardiovascular disease risk factors and demographic characteristics had an effect on sleep quality, it was found that age, gender, education level, and the presence of chronic disease did not predict sleep quality (Table SI).

## 4 Discussion

This study, conducted to determine the knowledge level about cardiovascular disease risk factors and the sleep quality of people with epilepsy, concluded that the patients’ cardiovascular disease knowledge level was above average, while their sleep quality was poor.

### 4.1 Cardiovascular Disease Risks in People With Epilepsy

Although the level of cardiovascular disease knowledge of the patients in this study was above average (Table 2), analyzing the descriptive characteristics of the patients revealed that they had cardiovascular disease risk factors such as “being overweight/obese, having diabetes mellitus Type 2” as stated in the American College of Cardiology (ACC) and American Heart Association (AHA) 2019 Cardiovascular Disease Prevention Guidelines (Arnett et al. 2019).

In this study, more than half of the patients were in the overweight and obese group (Table 1). Obesity is also an important issue in epilepsy and is commonly observed. Anti‐seizure medications, decreased physical activity, and dietary habits trigger the development of obesity in PWE (Li et al. 2024). In a cohort study of 554 newly diagnosed PWE aged between 16 and 87, it was reported that 55.2% of the patients were overweight and 31.2% were obese (Gaertner et al. 2024). An et al. (2014) found that the incidence of diabetes increased with advancing age in PWE. Similarly, it has been emphasized that PWE are more prone to obesity compared to healthy individuals, and timely recognition of cardiovascular risks in this group of patients is crucial (Li et al. 2024).

Half of the patients in this study had DM as an additional chronic disease (Table 1). It is widely known that diabetes increases the risk of cardiovascular disease. The incidence of diabetes increases with age in PWE (An et al. 2014). Arend et al. (2018) found that fasting glucose levels were higher in PWE compared to those without epilepsy. In PWE, high cortisol levels, along with hypercortisolism, lead to the development of dyslipidemia, insulin intolerance, physical inactivity, and obesity, which in turn trigger the formation of diabetes (Li et al. 2021).

Studies focused on determining the cardiovascular disease risks in patients are limited. Terman et al. (2021) reported that PWE have a 52% higher cardiovascular disease risk compared to people without epilepsy. Also, in Sravanthi et al.’s (2021) study, blood pressure and heart rate were reported to be higher in PWE than in healthy individuals.

Anti‐seizure medications used for reducing the frequency and severity of seizures increase the risk of cardiovascular diseases (Lee‐Lane et al. 2021). In this study, the anti‐seizure medications used by the patients were “levetiracetam, carbamazepine, and sodium valproate” (Table 1). Studies have suggested that the use of carbamazepine and valproate increases the tendency for high plasma lipid levels, accelerating atherosclerosis (Pang et al. 2025), while the use of levetiracetam has been reported to prolong the QT interval (Nei et al. 2025). It has particularly been emphasized that valproate is associated with metabolic syndrome, which can deteriorate cardiovascular health through various mechanisms such as chronic obesity, hypertension, insulin resistance, and dyslipidemia (Abbasi et al. 2025). Considering the medications used and their effects, patients are at risk for cardiovascular diseases. Lifestyle behaviors, anti‐seizure medication use, and the presence of additional chronic diseases in PWE increase their cardiovascular disease risks.

### 4.2 Sleep Quality in People With Epilepsy

This study found that the sleep quality of PWE was poor (Table 3). When examining previous studies, similar results have been observed (Akdağ et al. 2024; Alenizi et al. 2023; Çilliler and Güven 2020; Quigg et al. 2016).

Although the knowledge scale regarding cardiovascular risk factors does not directly include information related to sleep, it may indirectly contribute to better sleep quality through its impact on health‐related behaviors. Given the reciprocal interaction between sleep and neurological health in patients with epilepsy, this relationship is considered particularly important (Shin and Bin 2025). Furthermore, due to the cross‐sectional design of this study, causal inferences cannot be made (Wang and Cheng 2020). The direction of the relationship remains unclear, and it is possible that individuals with better sleep quality are more likely to engage in health‐promoting behaviors and acquire more health‐related knowledge. Sleep plays a critical role in disease management, especially in patients with epilepsy, and future intervention studies are needed to elucidate the underlying mechanisms of this relationship in patients with epilepsy.

On the other hand, when evaluating the results of studies conducted in different populations since no similar study findings with PWE were available, it appears that insomnia is associated with both cardiovascular disease (CVD) risk and CVD mortality. A meta‐analysis highlighted that insomnia was associated with a 45% increased risk of developing CVD or dying from CVD during a 3 to 20‐year follow‐up of 122,501 individuals initially free of CVD (Javaheri and Redline 2017). Another meta‐analysis of 17 cohort studies involving 311,260 individuals without CVD at baseline reported that CVD mortality increased by 33% among those experiencing insomnia (Javaheri and Redline 2017). Additionally, another study found that people with insomnia had a higher risk of myocardial infarction after a ten‐year follow‐up (Hsu et al. 2015).

### 4.3 Other Findings

In this study, women's total scores on the cardiovascular disease risk factors knowledge scale were higher compared to men's (Table 4). Cardiovascular diseases are among the leading causes of death in women (Kayikcioǧlu and Oto 2020). One study noted that women had a higher likelihood of benefiting from health education and resources than men (Wenger et al. 2022), while another study reported that women had greater awareness of CVD risk factors (Vogel et al. 2021).

Additionally, married individuals’ subscale scores for cardiovascular disease risk factors knowledge level were higher than those of single individuals (Table 4). Marital status has been listed among the factors that affect health behaviors, with being single, divorced, or widowed negatively impacting health behaviors (Bolton and Rodriguez 2009).

In our study, the sub‐dimension scores of the knowledge level of change in risk behaviors of those with generalized epilepsy type were higher than the sub‐dimension scores of the knowledge level of change in risk behaviors of those with focal epilepsy type (Table 4). The loss of consciousness of individuals during generalized seizures may increase the probability of these individuals encountering risky situations in their daily lives. This added risk may require them to pay more attention to protecting their health (Tülek 2021). Indeed, generalized epilepsy was more frequent in this study (Table 1).

Besides, we found that individuals with comorbid conditions had higher total cardiovascular disease risk factor knowledge scores and subscale scores for cardiovascular disease characteristics knowledge than those without comorbid conditions (Table 4). In our study, among people with epilepsy, 50.8% had diabetes (Table 2). The risk of diabetes is higher in people diagnosed with epilepsy compared to the general population. Furthermore, it is well known that diabetes increases the risk of cardiovascular disease (Gaertner et al. 2024; Terman et al. 2021). Nearly all of our patients have been diagnosed with epilepsy for more than ten years. Individuals with chronic diseases may feel a greater need to acquire more information about their health status, which could increase their awareness of managing cardiovascular health risks. Moreover, we believe that regular health checks and follow‐up processes ensured that these individuals were knowledgeable about cardiovascular disease risk factors. Patients’ knowledge scores about cardiovascular disease risk factors (smoking, diet, salt consumption, exercise, stress, presence of DM, HT, and blood lipid levels) were above average. However, it is inevitable for the patients to be in the risk group for cardiovascular disease and to have poor sleep quality due to their epilepsy, chronic comorbidities, anti‐seizure medications, seizures, and lifestyle behaviors. Since studies on this topic are limited, the discussion of research findings has been restricted. It is important to monitor the implementation of behaviors that will promote healthy lifestyle habits and keep cardiovascular disease risk factors under control in people with epilepsy. There is a need to employ epilepsy case manager nurses who will closely monitor patients in this area. A multidisciplinary team approach to patient follow‐up is crucial.

## 5 Conclusion and Recommendation

Half of the people with epilepsy had poor sleep quality. Knowledge level of cardiovascular disease risk factors was above average.

It is important for healthcare professionals, especially nurses who spend the longest time with the patient, to educate epilepsy patients about cardiovascular disease risk factors. Sleep quality should be evaluated periodically, and patients with poor sleep quality should be given sleep hygiene training.

### 5.1 Limitations

The fact that the study was conducted in a single center and that the findings were based on the patients' subjective statements constitute the study's limitations.

### 5.2 Strengths

This is the first study to evaluate the level of knowledge of cardiovascular disease risk factors and sleep quality together in people with epilepsy.

## Author Contributions

**Heycan Erdoğan**: writing – review and editing, writing – original draft, validation, visualization, software, supervision, resources, methodology, funding acquisition, formal analysis, conceptualization, data curation. **Hatice Karabuğa Yakar**: writing – review and editing, writing – original draft, validation, supervision, project administration, resources, methodology, investigation, funding acquisition, formal analysis, data curation, conceptualization.

## Funding

The authors have nothing to report.

## Consent to Participate

All participants provided their informed consent prior to participating in the study.

## Conflicts of Interest

The authors declare that they have no known competing financial interests or personal relationships that could have appeared to influence the work reported in this paper.

## Supporting information




**Supporting Information**: brb371573‐sup‐0001‐SuppMat.doc

## Data Availability

Data will be made available on request
