# Supplementary material for: Knowledge Level of Cardiovascular Disease Risk Factors and Sleep Quality in People With Epilepsy
Source: Brain Behav. 2026 Jul 8;16(7):e71573. doi: 10.1002/brb3.71573 (PMC13344888; doi:10.1002/brb3.71573)

**SUPPLEMENTARY FİLES**

**Appendix 1.** Statistical Notes

Prior to conducting the regression analyses, several variable transformations and coding procedures were applied. The number of anti-seizure medications (ASM count) was computed by summing the presence (coded as 1 = "Yes") of eight anti-seizure drugs (sodium valproate, levetiracetam, carbamazepine, lacosamide, oxcarbazepine, lamotrigine, phenytoin sodium, and topiramate) for each participant, yielding a continuous variable ranging from 0 to 8. Seizure frequency was operationalized from the ordinal variable "number of seizures in the past month" (0 = none, 1 = 1–3, 2 = 3–5, 3 => 5) and entered as a continuous predictor in the primary model. Categorical variables were dummy-coded: gender (0 = female, 1 = male), education level with "Primary-Middle School" as the reference category (creating three dummy variables for Literate, High School, and University), and epilepsy type (0 = focal, 1 = generalized). Age was included as a continuous variable.

Given that seizure frequency is a 4-category ordinal variable, the method recommended by Harrell (2015) (42) was employed to assess the statistical adequacy of modeling this variable as a continuous predictor with a linear trend. Specifically, the linear (original) coding of the variable was entered into the model alongside k−2 (4−2=2) dummy variables to test this assumption. The analysis indicated no significant departure from linearity (Wald
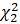
 = 4.06, p = 0.131). This finding supports the adequacy of treating seizure frequency as a continuous predictor in the primary model.The linearity assumption for age was visually examined using locally weighted scatterplot smoothing (LOWESS) curves with 95% confidence bands (span = 0.75). The LOWESS curve closely followed the linear regression line (Δ*R*² = 0.01), supporting the appropriateness of modeling the age as a linear predictor. The association between age and sleep quality was non-significant (*p* = .59), indicating a minimal confounding effect.

Before interpreting the regression results, several assumptions underlying the multiple linear regression were examined. The normality of the residuals was assessed using the Shapiro-Wilk test and visual inspection of the Q-Q plots. The test indicated a statistically significant deviation from normality, *W* = 0.97, *p* < .001. The Q-Q plot revealed slight deviations at both tails of the distribution, with the residuals falling below the reference line at the lower end and above the upper end. However, given the large sample size (*N* = 303), the central limit theorem suggests that the regression estimates remain robust despite violations (43). Homoscedasticity was evaluated using the Breusch-Pagan test and visual examination of the residuals versus the fitted values plot. The test was non-significant, *BP*(8) = 10.98, *p* = .20, indicating that the assumption of homoscedasticity was met. The scatterplot showed no systematic pattern or funnel shape, with residuals randomly distributed around the horizontal line at zero. Multicollinearity was examined using Variance Inflation Factors (VIF) and variable clustering analysis. All VIF values ranged from 1.03 to 1.34, well below the conventional threshold of 5 (or even the more conservative threshold of 10), indicating no multicollinearity among the predictors. The variable clustering dendrogram using Spearman ρ² showed minimal clustering, confirming that the predictors were sufficiently independent.

Influential observations were identified using Cook's distance with a threshold of 4/N = 0.01. A Cook's distance plot was inspected to visually identify influential observations (Figure 2). Eight observations (cases 2, 7, 8, 13, 30, 273, 285, and 287) exceeded this threshold value. To assess the impact of these outliers on the model estimates, a robustness check was conducted by comparing the full model (N = 303) with a model that excluded outliers (N = 295). The primary predictor (KARRIF) showed negligible differences in regression coefficients (β difference = 0.00) and maintained statistical significance in both models (p < .001), demonstrating that the model results were robust to influential observations. Linearity and potential model misspecification were assessed using Ramsey's Regression Equation Specification Error Test (RESET) and component-plus-residual (partial residual) plots. The RESET test was non-significant, F(2, 291) = 0.83, p = .437, supporting the linearity assumption. Additionally, the linearity of the relationship between age and sleep quality was visually evaluated using a scatterplot with a LOESS smoother and a linear regression line (Figure 3). As shown in Figure 3, the LOESS curve closely followed the linear regression line without clear systematic curvature, suggesting that modeling age as a linear predictor is appropriate.

In summary, while the normality assumption was violated, all other regression assumptions were adequately met, and the model demonstrated robustness against outliers. The large sample size provides confidence in the validity of regression estimates.

**Appendix II.**

**Figure S I.**

Cook’s Distance Plot for Detecting Influential Observations.


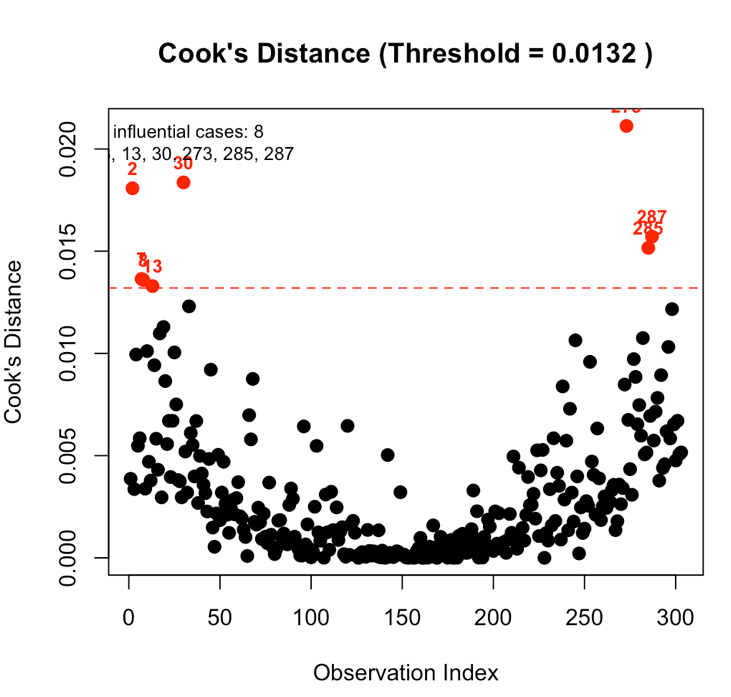


**Appendix III.**

**Figure S II.** Relationship Between Age and Sleep Quality


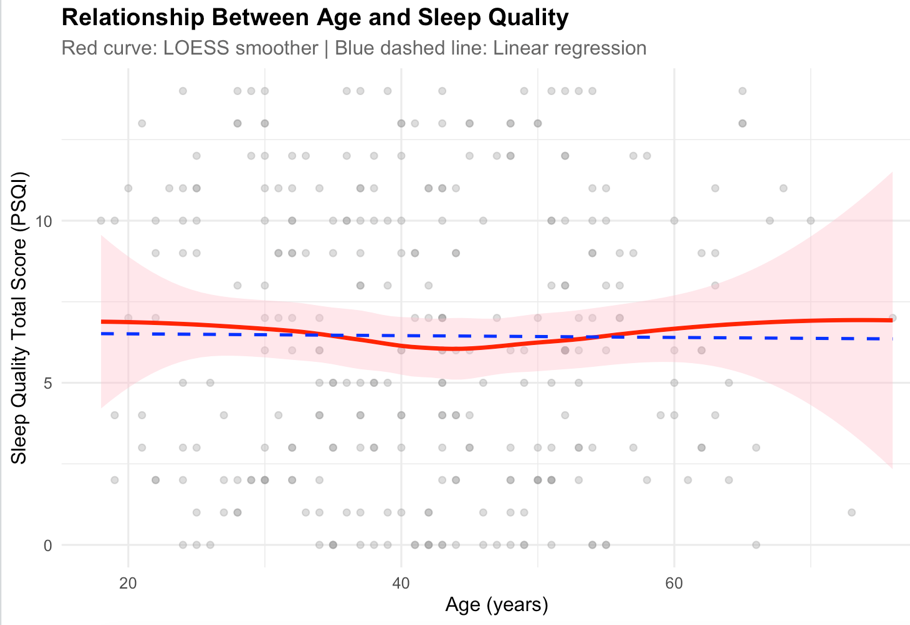


**Appendix IV.**

**Table S I:** The Effect of Cardiovascular Disease Risk Factors Knowledge Level and Some Characteristics Features on Pittsburgh Sleep Quality in People with Epilepsy (N=303)

| **Variable** | ***B*** | ***SE*** | **β** | ***t*** | ***p*** | **95~~.00~~% CI** | VIF |
| --- | --- | --- | --- | --- | --- | --- | --- |
| **Model 1** |  |  |  |  |  |  |  |
| **Constant** | 13.97 | 1.72 | 0.00 | 8.14 | < .001 | 10.59-17.35 |  |
| Cardiovascular Disease Risk Factors KnowledgeTotal Score | -0.39 | 0.08 | -0.28 | -5.01 | < .001 | -0.54—0.24 | 1.001 |
| Age | 0.0006 | 0.02 | 0.002 | 0.03 | .978 | -0.04-0.04 | 1.001 |

F(2,300)= 12.58; *p* < .001, *R*2 = .0.08

| **Variable** | ***B*** | ***SE*** | **β** | ***t*** | ***p*** | **95~~.00~~% CI** |  |
| --- | --- | --- | --- | --- | --- | --- | --- |
| **Model 2** |  |  |  |  |  |  |  |
| **Constant** | 13.74 | 1.74 | 0.00 | 7.89 | < .001 | 10.31-17.17 |  |
| Cardiovascular Disease Risk Factors KnowledgeTotal Score | -0.38 | 0.08 | -0.27 | -4.91 | < .001 | -0.54—0.23 | 1.009 |
| Educational status (primary-secondary school) | 0.43 | 1.04 | 0.05 | 0.41 | .680 | -1.62-2.49 | 1.009 |
| Educational status (high school) | 0.13 | 1.09 | 0.01 | 0.12 | .902 | -2.00-2.27 | 1.009 |
| Educational status (university) | -0.57 | 1.13 | -0.05 | -0.50 | .615 | -2.78-1.65 | 1.009 |

F(4,298)= 6.90; *p* < .001, *R*2 = .08

| **Variable** | ***B*** | ***SE*** | **β** | ***t*** | ***p*** | **95~~.00~~% CI** |  |
| --- | --- | --- | --- | --- | --- | --- | --- |
| **Model 3** |  |  |  |  |  |  |  |
| **Constant** | 14.28 | 1.58 | 0.00 | 9.07 | < .001 | 11.18-17.38 |  |
| Cardiovascular Disease Risk Factors KnowledgeTotal Score | -0.40 | 0.08 | -0.28 | -5.01 | < .001 | -0.55—0.24 | 1.018 |
| Gender | -0.35 | 0.49 | -0.0  4 | -0.71 | .476 | -1.31-0.61 | 1.018 |

F(2,300) = 12.85, p < .001, R^2^ = .08

| **Variable** | ***B*** | ***SE*** | **β** | ***t*** | ***p*** | **95~~.00~~% CI** | **VIF** |
| --- | --- | --- | --- | --- | --- | --- | --- |
| **Model 4** |  |  |  |  |  |  |  |
| **Constant** | 13.86 | 4.43 | 0.00 | 3.13 | 0.003 | 4.99-22.74 |  |
| Cardiovascular Disease Risk Factors KnowledgeTotal Score | -0.49 | 0.20 | -0.32 | -2.43 | 0.018 | -0.90—0.09 | 1.153 |
| Presence of diabetes | 1.34 | 1.30 | 0.15 | 1.03 | 0.310 | -1.28-3.95 | 1.429 |
| Presence of asthma | 2.68 | 1.60 | 0.23 | 1.68 | 0.10 | -0.53-5.89 | 1.212 |
| Presence of hyperthyroidism | 2.85 | 1.41 | 0.28 | 2.02 | 0.048 | 0.02-5.67 | 1.266 |

F(4,54) = 2,99, p < .001, R^2^ = .18

**Appendix V.**

**Table S II**. Multiple Linear Regression Analysis Results: Predictors of Sleep Quality

| Variable | Coeff | SE | β | t | p |
| --- | --- | --- | --- | --- | --- |
| (Intercept) | 15.33 | 2.02 | — | 7.58 | < .001 |
| KARRIF | -0.39 | 0.08 | -0.28 | -4.86 | < .001 |
| Age | -0.01 | 0.02 | -0.03 | -0.54 | 0.59 |
| Gender | -0.35 | 0.50 | -0.04 | -0.70 | 0.49 |
| Education (Literate) | -0.52 | 1.06 | -0.03 | -0.49 | 0.62 |
| Education (High School) | -0.35 | 0.60 | -0.04 | -0.59 | 0.55 |
| Education (University) | -1.14 | 0.70 | -0.10 | -1.62 | 0.11 |
| Number of ASMs | -0.13 | 0.35 | -0.02 | -0.38 | 0.71 |
| Seizure Frequency | 0.03 | 0.26 | 0.01 | 0.10 | 0.92 |
| Epilepsy Type | -0.22 | 0.72 | -0.02 | -0.30 | 0.77 |

*Note.* *N* = 303. *R*² = .088, Adjusted *R*² = .060, *F*(9, 293) = 3.15, *p* = .00, Cohen's *f*² = .10 (small effect). KARRIF: Cardiovascular Disease Risk Factor Knowledge Total Score. Education reference category: Primary–middle school. Sex reference category: Female. Epilepsy Type reference category: Focal. ASMs: Anti-seizure Medications.

**Appendix VI.**

Table S III. Correlation Analysis Between the Level of Knowledge of Cardiovascular Disease Risk Factors and Pittsburgh Sleep Quality Total Scores in People with Epilepsy

| **Pittsburgh Sleep Quality Index Total Score** |  | **Cardiovascular Disease Risk Factors KnowledgeTotal Score** | **Cardiovascular Disease Characteristics Subscale Score** | **Cardiovascular Risk Factors Subscale Score** | **Change in Risk Behaviors Subscale Score** |
| --- | --- | --- | --- | --- | --- |
|  | r | -0,27** | -0,14* | -0,29** | -0,09 |
|  | p | 0,00 | 0,01 | 0,00 | 0,10 |

*<0,05; **<0,01; r: Pearson Correlation

**Appendix VII. Results Notes**

To identify predictors of sleep quality while controlling for demographic and clinical covariates, a standard multiple regression analysis was performed. The results are presented in Table S II.

Table S II shows that multiple linear regression analysis revealed that cardiovascular disease risk factor knowledge (KARRIF) was the only statistically significant predictor of sleep quality among all variables examined. Specifically, for each one-unit increase in the KARRIF score, sleep quality scores decreased by 0.39 points, *t*(293) = -4.86, *p* < .001, indicating that better cardiovascular risk knowledge was associated with better sleep quality (lower PSQI scores). In contrast, no demographic or clinical covariates reached statistical significance. Age showed a negligible, non-significant association with sleep quality (*Coeff* = -0.01, *t* = -0.54, *p* = .59), suggesting that each additional year of age was associated with only a 0.01-point decrease in sleep quality score. Similarly, male sex was associated with a non-significant 0.35-point decrease in sleep quality scores compared to females (*Coeff* = -0.35, *t* = -0.70, *p* = .49).

Regarding education level, when compared to the reference category (Primary-Middle School), being literate was associated with a non-significant 0.52-point decrease (*Coeff* = -0.52, *t* = -0.49, *p* = .62), high school graduation with a non-significant 0.35-point decrease (*Coeff* = -0.35, *t* = -0.59, *p* = .55), and university graduation with a non-significant 1.14-point decrease (*Coeff* = -1.14, *t* = -1.62, *p* = .11) in sleep quality scores. Clinical variables also showed no significant associations: the number of antiseizure medications (*Coeff* = -0.13, *t* = -0.38, *p* = .71), seizure frequency (*Coeff* = 0.03, *t* = 0.10, *p* = .92), and having generalized epilepsy type versus focal (*Coeff* = -0.22, *t* = -0.30, *p* = .77) were all non-significant predictors. The overall model was statistically significant, *F*(9, 293) = 3.15, *p* = .001, explaining 8.8% of the variance in sleep quality (*R*² = .088; adjusted *R*² = .060). The effect size was small (Cohen's *f*² = .10), indicating that although the model reached statistical significance, the predictors accounted for a modest proportion of variance in sleep quality outcomes. Cardiovascular disease risk factors knowledge level was found to be negatively associated with the sleep quality scores of epilepsy patients (Table S III).


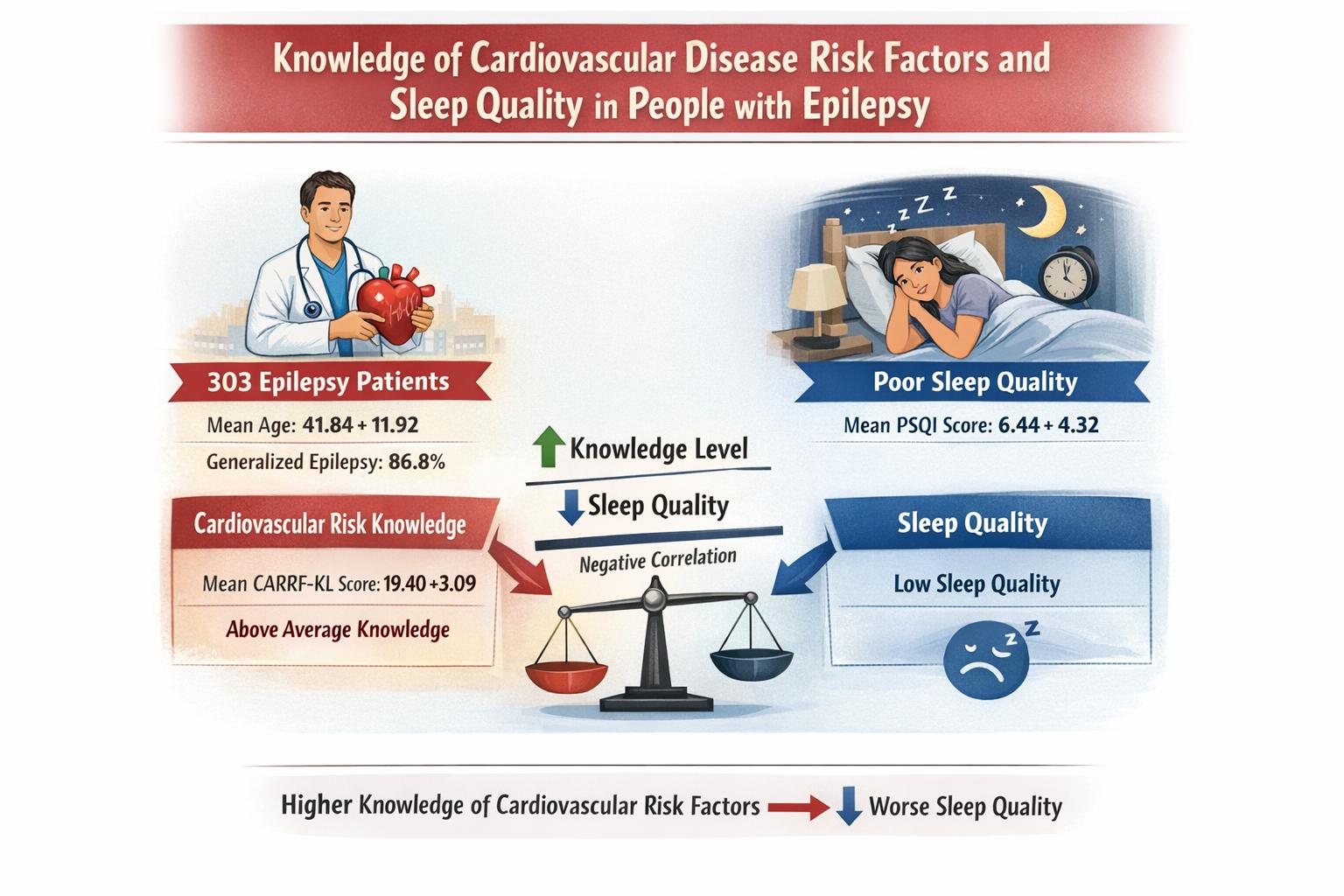

Supplement: Supplementary file 1 — Supporting Information: brb371573‐sup‐0001‐SuppMat.doc [file BRB3-16-e71573-s001.doc]
